# Effects of Coffee on Sirtuin-1, Homocysteine, and Cholesterol of Healthy Adults: Does the Coffee Powder Matter?

**DOI:** 10.3390/jcm11112985

**Published:** 2022-05-25

**Authors:** Gustavo Henrique Ferreira Gonçalinho, José Rafael de Oliveira Nascimento, Bruno Mahler Mioto, Reynaldo Vicente Amato, Miguel Antonio Moretti, Célia Maria Cassaro Strunz, Luiz Antonio Machado César, Antonio de Padua Mansur

**Affiliations:** 1 Faculdade de Medicina, Universidade de Sao Paulo, Sao Paulo 01246-903, SP, Brazil; gustavo.goncalinho@usp.br (G.H.F.G.); jrafaelno@usp.br (J.R.d.O.N.); dcllucesar@incor.usp.br (L.A.M.C.); 2 Servico de Prevencao e Reabilitacao Cardiovascular, Instituto do Coracao (InCor), Hospital das Clinicas HCFMUSP, Faculdade de Medicina, Universidade de Sao Paulo, Sao Paulo 05403-900, SP, Brazil; 3 Unidade Clinica de Coronariopatias Cronicas, Instituto do Coracao (InCor), Hospital das Clinicas HCFMUSP, Faculdade de Medicina, Universidade de Sao Paulo, Sao Paulo 05403-900, SP, Brazil; brunomioto@gmail.com (B.M.M.); reynaldo.amato@incor.usp.br (R.V.A.); mamorett@uol.com.br (M.A.M.); 4 Laboratorio de Analises Clínicas, Instituto do Coracao (InCor), Hospital das Clinicas HCFMUSP, Faculdade de Medicina, Universidade de Sao Paulo, Sao Paulo 05403-900, SP, Brazil; labcelia@incor.usp.br

**Keywords:** coffee, cardiometabolic health, bioactive compounds, sirtuin-1, cholesterol, homocysteine, Arabica, Robusta

## Abstract

Background: Coffee is one of the most popular beverages globally and contains several bioactive compounds that are relevant to human health. Many nutritional strategies modulate sirtuin-1, thereby impacting aging and cardiometabolic health. This study investigated the influence of different blended coffees on serum sirtuin-1, blood lipids, and plasma homocysteine. Methods: An eight-week randomized clinical trial that included 53 healthy adults of both sexes analyzed the effects of daily intake of 450 to 600 mL of pure Arabica or blended (Arabica + Robusta) coffee intake of filtered coffee on blood sirtuin-1, lipids, and homocysteine. Results: Both Arabica and blended coffees similarly increased serum sirtuin-1 concentration, from 0.51 to 0.58 ng/mL (*p* = 0.004) and from 0.40 to 0.49 ng/mL (*p* = 0.003), respectively, without changing plasma homocysteine, folic acid, glucose, and CRP. However, the blended coffee intake increased total cholesterol from 4.70 to 5.17 mmol/L (*p* < 0.001) and LDL-cholesterol from 2.98 to 3.32 mmol/L (*p* < 0.001), as well as HDL-c from 1.26 to 1.36 mmol/L (*p* < 0.001). Conclusion: Both coffee powders increased sirtuin-1 expression, but our results suggest that blended coffee had hypercholesterolemic effects which could increase cardiovascular risk. Therefore, preference should be given to Arabica coffee for the best cardiometabolic benefits of coffee.

## 1. Introduction

Sirtuins are class-III NAD+-dependent deacetylases that have gained prominence in scientific literature due to their health effects, being a potential therapeutic target for several pathologies [1]. In humans, seven isoforms of sirtuins (sirtuin-1 to sirtuin-7) are distributed in cell cytoplasm, nucleus, and mitochondria [1]. Sirtuins possess deacetylase activity and also ADP-ribosyl-transferase activity and regulate the activity of several transcriptional factors besides several targets [2]. Sirtuins have a wide range of roles in immunometabolic homeostasis, and sirtuin-1 (Sirt-1), which is found mainly in the cytoplasm and nucleus, is the most studied.

Studies showed that intracellular sirtuins are involved in longevity [3]. Sirt-1 improved telomere length maintenance in vivo and significantly increases recombination frequencies at telomeres, centromeres, and chromosome arms and DNA repair efficiency through deacetylation of nibrin (Nbs1) and WRN helicase, two important players in DNA repair by homologous recombination [4]. These effects are beneficial to preserve genome integrity and stability, leading to increased healthspan, and protection from some age-associated diseases [4]. In experimental studies, the Sirt-1 agonist resveratrol increases the life expectancy of many species [3]. Nevertheless, little is known concerning the Sirt-1 serum concentrations. Dietary energy restriction also increased life expectancy [5], and higher Sirt-1 serum concentration could be related to this outcome. Calorie restriction increased Sirt-1 serum concentrations and gene expression in clinical studies [6,7]. It was also found that dietary energy restriction decreases plasma noradrenaline level, which is increased in aging and cardiometabolic diseases, showing a potential relationship between Sirt-1 and the neurotransmitter [8]. However, energy was severely restricted in these studies, which could lead to a long-term risk for other complications [6,7,8]. Thus, studies showing calorie restriction-mimetics substances such as resveratrol have gained attention [9].

Further evidence showed that loss of function of *SIRT1* is implicated in several pathological processes, such as endothelial dysfunction, atherosclerosis, cardiovascular diseases, hypercholesterolemia, hypertension, diabetes mellitus, obesity, among others [1]. Sirt-1 activation restored mitochondrial function, a hallmark of endothelial function and aging-related diseases [10]. Moreover, the pro-inflammatory state of non-communicable diseases decreased Sirt-1, and its activation mitigated inflammation [11].

Several compounds activate Sirt-1, many of them found in foods [6,7,8,12,13]. Coffee is one of the most consumed beverages globally and moderate consumption (3 to 5 cups) is associated with cardiovascular protection [14,15]. Coffee compounds, such as chlorogenic acid (CGA), caffeine, trigonelline, melanoidines, cafestol, and kahweol, prevented free radical damage, inhibited inflammatory mediators release, improved lipid and glucose metabolism, and promoted vasodilation [16]. The metabolic coffee effects were similar to those observed with Sirt-1 activation [1]. Therefore, it is feasible that coffee consumption may interact with Sirt-1 expression.

Nevertheless, different coffee species, blends, and brewing methods vary in composition of bioactive compounds, which could have different effects on human metabolism. The most cited variation in health effects of types of coffee is concerning filtration. Filters reduce diterpene levels, which have hypercholesterolemic effects and potentially increase cardiovascular risk [17]. Differences in coffee composition were also shown in different species. Robusta (*Coffea canephora*) contains more caffeine than Arabica (*Coffea arabica*) [18,19], while the latter has a higher content of diterpenes [20]. Previous studies showed that polyphenols, antioxidant activity, and metabolites, such as CGA, trigonelline, and choline, were higher in both green and roasted coffee belonging to the Arabica species, regardless of the extraction procedure, roasting process, and geographical origin compared to Robusta species [19,21,22]. Other reports showed that Arabica and Robusta species had similar ROS-reducing potential, but the roast degree of the beans seems to be the decisive influential factor on antioxidant effect [23].

Although studies suggest that the effects of coffee consumption vary depending on the species, origin, roast degree, and form of preparation, there is still a lack of studies comparing the effect of different coffees on human physiology. Furthermore, the consumption of different coffee powders may be responsible for the inconsistent results found in the literature regarding cardiometabolic effects. In our study, therefore, we investigated the effects of two different popular coffee powders on serum sirtuin-1, blood lipids, and plasma homocysteine of healthy adults.

## 2. Materials and Methods

### 2.1. Study Design, Participants, and Intervention

**Figure 1:**
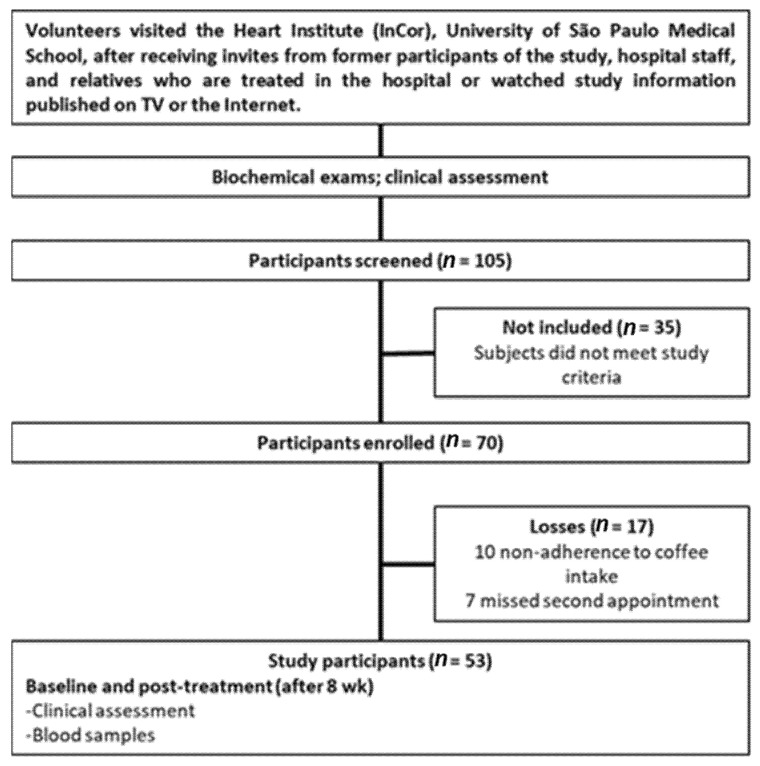
Flow diagram of participant progress through the study.

This was an eight-week parallel randomized clinical trial derived from the study “Influence of Coffee on the Health and on the Heart of Normal, Diabetic, and Coronary Artery Disease Patients” (trial registration number: NCT01003392). In the present study, 53 healthy adults (13 men and 40 women) aged from 20 to 70 years old participated. The study investigated the effects of 450 to 600 mL (3 to 4 cups) of daily coffee intake containing 100% Arabica (*Coffea arabica*) powder or a blended powder with 80% Arabica and 20% Robusta (*Coffea canephora*) on Sirt-1 (Figure 1). As secondary outcomes, blood lipids and homocysteine were evaluated. Both coffee interventions were chosen because *Coffea arabica* and *Coffea canephora* are the species that have the highest commercial value, being the most produced species worldwide [24,25]. The aroma and flavor of Arabica coffee are more appreciated than Robusta coffee, but the price is more expensive. Blends using mixed Arabica and Robusta powders are cheaper and are widely consumed [26]. The differences in the chemical composition of Arabica and Robusta powders are described in Appendix A Table A1. This characterization was done in a previous study [27], which used commercial products of brands associated with the Brazilian Coffee Industry Association (ABIC), such as the coffee powders used in the present study (provided by Melitta do Brasil Indústria e Comércio Ltda., São Paulo, Brazil).

The participants were healthy volunteers, and the study was developed at the Instituto do Coracao (InCor) do Hospital das Clinicas HCFMUSP da Faculdade de Medicina da Universidade de Sao Paulo, Sao Paulo, SP, Brazil. Inclusion criteria consisted of healthy volunteers with normal physical examination and electrocardiogram. The exclusion criteria were smoking, alcohol abuse, use of medications or supplements, hypertension, dyslipidemia diabetes hormone replacement therapy, non-adherence to proposed intervention (i.e., not drinking coffee for a day during study time), and chronic non-communicable diseases. Hypertension was defined as the use of antihypertensive medication or self-reported hypertension. Dyslipidemia was defined as the use of lipid-lowering medication, serum triglyceride concentrations ≥ 1.69 mmol/L, or total cholesterol ≥ 6.21 mmol/L. Diabetes was defined as fasting blood glucose >7 mmol/L or the use of hypoglycemic medication.

The variables analyzed included age, body weight, blood lipids (triglycerides, total cholesterol, HDL-cholesterol, LDL-cholesterol, and lipoprotein (a) [Lp(a)]), glucose, insulin, high-sensitivity C-reactive protein (hs-CRP), homocysteine, folic acid, and Sirt-1. We calculated the homeostasis model assessment for insulin resistance (HOMA-IR) using the following equation: fasting insulin (µIU/mL) × fasting glucose (mmol/L)/22.5.

Two commercially available coffees (100% *Coffea arabica* and 80%/20% *Coffea arabica* and *Coffea canephora*) of caffeinated, roasted, ground coffee were used in the study. Both coffees were cultivated in the same geographic area. They were vacuum-packed in aluminized bags (500 g). The commercial coffees, coffee packages, and paper filters (Classic nº 102) were provided by Melitta do Brasil Indústria e Comércio Ltda., São Paulo, Brazil.

After a 21-days washout (without any use of caffeine-containing beverages or coffee intake), participants underwent a standardized interview and blood sample collection after a 12 h fast, which were repeated at the end of the study. Arabica or blended coffee powders were given to participants, who were instructed to consume between 450 to 600 mL daily of filtered coffee (15 g of coffee per one 150 mL of water) without a fixed schedule for eight weeks. During the study, consuming foods such as cocoa, guarana, chocolate, and tea was forbidden. Participants were also instructed to keep their routine dietary and physical activity habits. All participants reported adherence higher than 95% of the intervention time (i.e., at most 3 days of coffee consumption below the dose recommended by the researchers).

All subjects provided written informed consent. The research protocol was approved by the Ethics Committee of the University of São Paulo Medical School, Brazil (CAPPesq-879/04) and was in line with the Declaration of Helsinki.

### 2.2. Biochemical Analyses

Venous blood was drawn after 12 h of fasting. Serum total cholesterol, triglycerides, HDL-c, and glucose were obtained by commercial colorimetric-enzymatic methods. LDL-c was calculated using the Friedwald equation. Measurements were performed using Dimension RxL equipment (Siemens Healthcare Diagnostic Inc., Newark, DE, USA) with dedicated reagents. Lp(a) was obtained by immunonephelometry using dedicated reagents for BN-II equipment from Siemens Healthcare (Marburg, Hessen, Germany). Plasma total homocysteine, insulin and folic acid concentrations were measured by a chemiluminescence immunoassay on an Immulite 2000^®^ Analyzer (Siemens Healthcare Diagnostic Inc., Newark, DE, USA).

Serum Sirt-1 concentrations were determined using an ELISA kit (Uscn Life Science, Wuhan, Hubei, China). Sirt-1 samples, before and after interventions, were analyzed in duplicate and in the same ELISA plate and using the Multiscan FC plate reader (Thermo Fisher Scientific, Waltham, MA, USA), with a coefficient of variation of 12%, according to the manufacturer’s instructions. All analyses were performed according to manufacturers’ instructions.

### 2.3. Statistical Analyses

The sample size calculation was made by the difference between the serum Sirt-1 levels, before and after interventions [6,7]. The study had a randomized allocation of participants to the groups based on previous randomization sequences obtained from SPSS statistical software. Both participants and assessors were blinded for the group allocation. The difference between the mean values of Sirt-1 for Arabica coffee and blended coffee groups was 100 pg/dL with a standard deviation of 10 pg/dL. The test power (a priori) was β = 0.80 and α = 0.05 and a detection of 30% difference in serum Sirt-1 concentration was expected. Kolmogorov–Smirnov’s distribution test was used to assess variable distribution. Pre- and post-treatment variables were summarized with the use of descriptive statistics. Continuous parametric variables were summarized as mean and standard deviation (SD), whilst nonparametric variables were summarized as the median and interquartile range (IQR). Paired-sample *t*-test and Wilcoxon’s test were used for pre- and post-treatment comparisons. Independent *t*-test and Mann–Whitney’s U test were used for baseline, post-treatment, and variables’ changes comparisons between groups. Linear mixed models were applied to assess the effects of interventions on serum Sirt-1, plasma total cholesterol, LDL-c, and HDL-c (treatment and time as fixed effects), with all analyses adjusted by sex and age. The level of statistical significance was set at *p* < 0.05. The software used for statistical analysis was SPSS version 20.

## 3. Results

### 3.1. Baseline Data

The groups had predominantly female participants, with 75.8% and 75% in blended and Arabica coffee groups, respectively. The mean ages of blended and Arabica coffee groups were respectively 45.5 and 48.7 years (*p* = NS). All baseline parameters were similar among groups, except for plasma folic acid and serum Sirt-1, in which the Arabica group presented higher levels of both variables (Table 1). Overall serum concentration of Sirt-1 was 0.44 ± 0.13 ng/mL.

### 3.2. Post-Treatment Data

Post-treatment data and percentage mean change are described in Table 1 and Table 2, respectively. Overall serum concentration of Sirt-1 was 0.53 ± 0.16 ng/mL.

Post-treatment serum Sirt-1 was higher in Arabica coffee group (*p* = 0.043). However, Percentage mean changes of serum Sirt-1 increased approximately 15% in both groups (*p* = 0.846).

There was no difference in body weight of both groups at the end of the study. Total cholesterol increased 10.7% (*p* < 0.001) in the blended coffee group, whilst a non-significant 3.5% decrease was observed in the Arabica coffee group (*p* = 0.279). The percentage mean changes of total cholesterol differed significantly between both groups (*p* = 0.002). In the blended coffee group, HDL-c and LDL-c increased significantly by 8% (*p* < 0.001) and 12.7% (*p* < 0.001), respectively, after treatment. The percentage mean changes of HDL-c and LDL-c also differed significantly between both groups (*p* = 0.009 and *p* = 0.003, respectively).

At the end of the study, there were no statistically significant differences in triglycerides, glucose, insulin, HOMA-IR, hs-CRP, Lp(a), homocysteine, and folic acid in either group. Furthermore, there were no differences between groups in percentage mean change of these variables.

### 3.3. Adjusted Effects of Different Coffee Powders

The effects of both interventions adjusted by sex and age are outlined in Table 3 and Table 4.

Our findings show that both coffee powders had a significant effect on serum Sirt-1 (F[time] = 17.611, *p* < 0.001; F[coffee type] = 9.071, *p* < 0.001), marked with an increase (mean effect = 0.088 ng/mL, *p* < 0.001), that tended to be more pronounced in Arabica group (mean effect = 0.097 ng/mL, *p* = 0.004). However, interaction analysis showed no significant differences among groups at the end of the study (F[interaction] = 0.133, *p* = 0.717).

Interventions also increased LDL-c at the end of the study (F[time] = 5.950, *p* = 0.018; mean effect = 0.152 mmol/L, *p* = 0.018), with a significant time and coffee type interaction (F[interaction] = 8.992, *p* = 0.004), showing that LDL-c of the Arabica group tended to be 0.374 mmol/L lower at the end of the study (*p* = 0.004). Interaction analysis also showed that total cholesterol and HDL-c tended to be respectively 0.651 mmol/L (*p* = 0.001) and 0.121 mmol/L (*p* = 0.005) lower at the end of the study. However, no statistically significant mean effect was found in either parameter.

## 4. Discussion

This study showed that the consumption of 3 to 4 cups of blended (80% Arabica and 20% Robusta) or Arabica coffee increased the serum concentrations of Sirt-1. The study also showed that blended coffee increased serum cholesterol parameters, especially LDL-c while no differences were observed with pure Arabica consumption.

The literature’s most documented effects of sirtuins are associated with increased longevity, but decreased sirtuin expression and activity are also linked to the pathogenesis of cardiovascular and metabolic disorders [1]. It is clear that *SIRT1* knockout mice have decreased mitochondrial function, which can be recovered by NAD+ replenishment [10,11]. Moreover, chronic inflammation present in non-communicable diseases is accompanied by a state of low expression and activity of Sirt-1 [11]. However, the literature on the association of Sirt-1 serum concentrations with clinical outcomes is still scarce. He et al. [28] showed that serum Sirt-1 predicted high-risk coronary atherosclerotic plaque in individuals with low–intermediate Framingham Risk Score during computed tomography angiography. The hallmark of these conditions is the decline of mitochondrial function, which leads to a loss of cellular homeostasis and metabolic health.

Previous studies have shown that coffee and its by-products and bioactive compounds improve mitochondrial function and increase this organelle’s biogenesis [29,30,31,32,33]. One of the mechanisms was through the increase of PGC-1α expression, which is a key regulator of mitochondrial biogenesis and function [29]. It was demonstrated previously that Sirt-1 activation potently induced mitochondrial activity through PGC-1α activation [34]. Therefore, coffee could improve mitochondrial function through Sirt-1 modulation.

Despite many controversies, a large body of evidence suggests that coffee intake does not increase the risk of cardiovascular disease [14,15,35]. Furthermore, it was shown that moderate coffee consumption (3 to 5 cups) was a protective factor for overall cardiovascular diseases in a meta-analysis [14], which was corroborated by a more recent umbrella review of meta-analyses [15]. In a study that followed Mediterranean adults for 18 years, it was shown that, compared with no-consumption, low (1 or <1 cups) and moderate (2 to 6.5 cups) daily consumption were associated with a 27% and 44% reduction in all-cause mortality, respectively, whilst both doses were associated with 30% reduction in CVD mortality [36]. In the European Prospective Investigation into Cancer and Nutrition (EPIC) cohort which enrolled 521,330 persons within a mean follow-up of 16.4 years, it was shown that participants in the highest quartile of coffee consumption had statistically significant lower all-cause mortality in men and women (men: HR, 0.88 (95% CI, 0.82 to 0.95); *p* for trend < 0.001; women: HR, 0.93 (CI, 0.87 to 0.98); *p* for trend = 0.009) [37]. Among women, moderate to high consumption were also associated with cardiovascular mortality reduction [37]. Our study showed that moderate coffee consumption increased the serum concentrations of the cardioprotective protein Sirt-1. This result suggests that the cardiovascular risk reduction with moderate coffee consumption found in the studies mentioned above could also be related to increased Sirt-1 serum concentrations.

More than 14,000 sirtuin-activating compounds can modulate the Sirt-1 expression. Among these are natural compounds present in the diet, such as resveratrol, quercetin, myricetin, catechins, etc. [6,7,8,12,13]. Coffee beans have several compounds such as macronutrients, micronutrients, and hundreds of biologically active phytochemicals which affect cardiometabolic health. The coffee bioactivity is mainly attributed to chlorogenic acid (CGA), caffeine, trigonelline, melanoidines, cafestol, and kahweol [16]. Since our study has found that moderate coffee consumption increased serum Sirt-1, one or more of these compounds were likely involved in the modulation of Sirt-1.

The most studied coffee compounds are caffeine and CGA. CGA is the most abundant polyphenol in coffee. CGA has been shown to reduce oxidative damage, control the inflammatory response through inhibition of TNF-α, IL-6, and IL-1β, increase insulin sensitivity, and prevent CVD and obesity through lipid metabolism modulation [16]. A previous study has shown that pretreatment of human umbilical vein endothelial cells (HUVECs) and rat aortic endothelial cells with CGA effectively prevented oxLDL-impaired mitochondrial function and oxidative stress by modulating the Sirt-1/AMPK/PGC-1α pathway [38]. The authors also found that the increase of Sirt-1 activity by CGA treatment reduced oxLDL-induced endothelial apoptosis by preventing oxLDL-induced upregulation of Bax and inhibiting Bcl-2 [38]. Another study found that CGA attenuated vascular senescence in a dose-dependent association with increased Sirt-1 and eNOS expression and with the decrease of p-Akt, plasminogen activator inhibitor-1, p53, and p21 expression in HUVECs and aorta of mice infused with angiotensin II (AngII) [39]. It has also been found that the activation of Sirt-1 by CGA protects hepatocytes from free fatty acids-induced lipotoxicity in AML12 cells [40].

Regarding caffeine, few studies have shown the influence on sirtuins. Despite not having evaluated the Sirt-1 expression, previous studies have shown that caffeine increased yeast lifespan through TORC1 inhibition [41] and promoted the expression of telomerase reverse transcriptase gene (TERT) to delay cellular senescence and to age in addition to the restoration of organ decline in mice [42]. Sirtuins have been shown to play an essential role in these metabolic pathways [4,43,44]. Furthermore, caffeine is an agonist of SIRT3, increasing its antioxidant effects by superoxide dismutase 2 (SOD2) [45] and ameliorating lipid accumulation in the liver of high-energy diet-treated mice [46]. Together, these data corroborate our results, which showed that coffee consumption increased the serum concentration of Sirt-1. According to the mechanisms mentioned above, this could be attributed mainly to caffeine and CGA. However, the results of our study were not able to demonstrate this due to the lack of a measure of circulating caffeine and CGA.

More than 100 different species have been recognized so far, but *Coffea arabica* and *Coffea canephora* (known as Arabica and Robusta, respectively) represent the most relevant and widely cultivated species [26]. Arabica beans are more flavorful and expensive than Robusta, and commercial blends using both species are popular [19]. However, Arabica and Robusta may differ in composition, altering their biological effects on human health. It was shown that blends containing more Arabica beans had higher total phenolic compounds, mainly CGA, and antioxidant activity than 100% Robusta [19]. Another study showed that Arabica had higher 5-caffeoylquinic acid (5-CQA), the most abundant CGA in coffee, than Robusta [27]. Therefore, blends containing more Arabica could have better health effects. Regarding the increased Sirt-1 expression, however, no difference between the two groups was observed in our study. The increase in Sirt-1 could also be related to coffee components other than phenols.

Furthermore, our results have shown that the blended coffee group had a significant increase in total cholesterol, HDL-c, and especially LDL-c, even when adjusted by age and sex. Besides, there were differences in percentage changes of these parameters, although the LDL-c/HDL-c ratio was similar between groups. The blended coffee group increased by approximately 10% in these parameters, while no statistically significant differences were found in the Arabica group. Importantly, no differences in weight or body mass index after treatment were found in both groups, evidencing that the lipid profile modification was due to the interventions. Diterpenes, mainly cafestol and kahweol, present in coffee bean oil strongly influence lipid metabolism [47]. Diterpenes cause extracellular accumulation of LDL by reducing the activity of the LDL receptor, which is responsible for the endocytic process of apoB- and apoE-containing lipoproteins [48]. It has been suggested that coffee made from Robusta beans raises cholesterol more than coffee from Arabica beans [49]. However, it was found that Arabica oil has more diterpenes than Robusta oil, but both oils increased blood cholesterol equally in healthy individuals [20]. Although these hypercholesterolemic effects of diterpenes have been demonstrated, our study used filtered coffee, a brewing method that drastically reduces diterpenes levels [48]. Therefore, it is unlikely that the cholesterol increase observed in our study is due to the diterpenes in coffee.

The effect of coffee on HDL-c is still unclear, as shown in a meta-analysis of randomized controlled trials [17]. Our study showed an increase in HDL-c with blended coffee, indicating a potential increase in reverse cholesterol transport which could reduce cardiovascular risk. This is in line with a previous work that showed that caffeic acid and ferulic acids increased ABCG1 and SR-BI expression and enhanced cholesterol efflux from THP-1 macrophages mediated by HDL [50]. Therefore, simultaneous increases in LDL-c and HDL-c might neutralize the respective deleterious and beneficial effects of these particles on the cardiovascular system. However, no statistically significant mean effect was found in HDL-c, showing that this alteration in the lipid profile which increased LDL-c significantly might increase cardiovascular risk.

A recent meta-analysis of 12 randomized controlled trials has shown that coffee raises blood total and LDL-cholesterol in a dose-dependent manner [17]. It is important to note that no study evaluated differences between coffee species [17]. This result was found even in filtered coffee interventions but with a more discrete effect. Other randomized clinical trials have shown that filtered coffee increased total cholesterol, LDL-c, and HDL-c, and coffee abstention can reduce these parameters [51,52]. Moreover, in one of the studies mentioned, the same blended coffee containing 80% Arabica and 20% Robusta was used as in our study, and the amount of total cholesterol and LDL-c increase was similar to that observed in our study (about 0.47 mmol/L and 0.34 mmol/L, respectively) [52]. These data suggest that compounds other than diterpenes and blended coffee with Robusta have a hypercholesterolemic effect.

The reason that the intervention with blended coffee increased blood lipids could be attributed to caffeine. Robusta coffee species contain more caffeine than Arabica [18,19,53]. It has been previously shown, in vitro and in vivo using animal models, that caffeine supplementation increased hepatic cholesterol synthesis and serum cholesterol due to reduced incorporation of fatty acids and sterols in the liver [54]. Furthermore, prenatal caffeine exposure induces susceptibility to adult hypercholesterolemia in offspring rats [55]. The mechanism proposed was downregulation of *SIRT1* expression by inhibition of adenosine A2 receptor (A2AR)/cAMP/protein kinase A (PKA) pathway, thereby increasing the histone acetylation and cholesterol synthesis genes expression *SREBF2*, *HMGCR*, and *HMGCS1* that led to a hepatic cholesterol accumulation [55]. Interestingly, activation of Sirt-1 by an agonist (resveratrol) reversed the metabolic changes in adult offspring rats [55]. Furthermore, previous studies have also shown that caffeine ameliorates lipid metabolism and mitochondrial function through Sirt-3, which is correlated with Sirt-1 expression [45,46]. In our study, coffee increased Sirt-1 serum concentration, but it also increased total cholesterol, LDL-c, and HDL-c with blended coffee. Perhaps Sirt-1 activation was not enough to reverse the unfavorable metabolic effects of caffeine in our study. Further studies are required to understand which components of filtered coffee influence Sirt-1 and blood lipids serum concentrations.

Previous studies showed that homocysteine-induced upregulation of LOX-1 and type A and B endothelin are linked to Sirt-1 downregulation and activation of NF-κB [56,57,58,59]. However, we found no significant effects of coffee on homocysteine, despite the increased serum Sirt-1 at the end of the study. Further studies are needed to assess the effects of Sirt-1 and homocysteine.

Previous randomized clinical trials showed that coffee increased plasma homocysteine in healthy subjects, suggesting that coffee could increase cardiovascular risk [51,60,61,62]. On the other hand, abstention from filtered coffee decreased homocysteine plasma concentration [51]. It was suggested that caffeine is partially responsible for increased homocysteine by coffee intake [61], but another study showed it was associated with caffeic acid, one type of CGA, suggesting that type of coffee could influence homocysteine differently [62]. However, homocysteine did not change in our study. Furthermore, it is important to note that the increase in cardiovascular risk caused by coffee occurs only in heavy coffee drinkers [51,60,61,62] In addition, folic acid, the primary modifier of plasma homocysteine, remained unchanged at the end of the study. This finding is in line with the results of previous randomized clinical trials [51,62].

Our study has limitations. First, the small sample size is the main limitation for analyzing blood biomarkers, but not for Sirt-1 serum concentrations. Second, we did not assess the bioactive compounds of powder coffees studied. The lack of assessment of these bioactive compounds makes it impossible to attribute coffee-specific compounds’ effects accurately. The lack of a control group is another limitation that would exclude random variability in the studied outcomes. In addition, self-reported adherence to the study protocol is a limitation, because data reported by the subjects could be distorted.

This study is the first one that evaluated the different effects between Arabica and Arabica + Robusta coffees in Sirt-1 serum concentration in humans. Traditional risk factors explain most of the cardiovascular events [63]. However, emerging risk factors have gained emphasis and Sirt-1 has gained prominence as a marker of endothelial function and aging [64]. However, increased Sirt-1 concentration must be interpreted with caution because further studies are needed to investigate the relationship between serum Sirt-1 and clinical outcomes. Nevertheless, evidence points to associations of serum sirtuin-1 levels with protection against chronic diseases and markers of aging and endothelial dysfunction [28,65,66,67]. It was shown that although increased in Sirt-1 serum concentrations, serum cholesterol also raised in the blended coffee group. However, since the increase of Sirt-1 has been observed in both groups, while the cholesterol increase occurred only in the blended coffee group, it is suggested that the hypercholesterolemic effect is not correlated to Sirt-1. Total and LDL-cholesterol are well-known cardiovascular disease biomarkers, so the net effect on cardiovascular health needs further evaluation.

In addition, as far as we know, this was the first study that compared the effects of two specific popular blended coffees in various blood biomarkers, showing that the type of coffee may influence cardiovascular health in different ways. Limitations of the study include the small sample size and lack of analysis of coffee bioactive substances and their metabolites in the blood, requiring caution in interpreting our results. Future studies should consider in their analysis the effects of coffee species and mixtures on health. The lack of adequate studies is responsible for inconsistencies in the scientific literature about the effects of coffee consumption, given that most studies do not describe the coffee powder used.

## 5. Conclusions

Our study showed that daily moderate coffee intake (3 to 4 cups/d) increased Sirt-1 serum concentration, which could be one of the biomarkers responsible for the cardiovascular protection observed in previous epidemiological studies. However, our findings also showed distinct effects on blood lipids achieved by different coffee powders. Blended coffee powder presented a hypercholesterolemic effect, probably related to lower polyphenols and higher caffeine content in Robusta beans. According to the results of our study, coffee intake might bring cardiometabolic benefits due to Sirt-1 modulation, but a pure Arabica coffee powder could lead to optimal vascular health due to the hypercholesterolemic effect of the coffee blended with Robusta. As far as it is known, this was the first clinical study investigating the effect of different coffee powders on serum Sirt-1. Further studies should focus on different coffee powders and their respective effects on human health, exploring their effects on markers of mitochondrial function in healthy individuals and patients with cardiometabolic diseases.

## Figures and Tables

**Table 1:** Baseline and post-treatment characteristics of the participants.

	Blended Coffee Group				*p*	Arabica Coffee Group				*p*	Baseline *p*	Post-Treatment *p*
	Baseline		Final			Baseline		Final				
	*n* = 33		*n* = 33			*n* = 20		*n* = 20				
Variables	Mean	SD	Mean	SD		Mean	SD	Mean	SD			
Age, years	45.5	12.2	45.5	12.2	*N/A*	48.7	14.1	48.7	14.1	*N/A*	0.392	*N/A*
Sex (male/female), *n* (%)	8/25 (24.2/75.8)				*N/A*	5/15 (25.0/75.0)				*N/A*	N/A	*N/A*
Body weight, kg	68.0	10.3	67.9	10.2	0.523	69.0	11.5	68.7	11.2	0.230	0.755	0.784
Body mass index, kg/m^2^	26.9	4.1	27.0	4.1	0.230	26.2	3.9	26.1	3.8	0.132	0.517	0.399
Total cholesterol, mmol/L	4.70	0.79	5.17	0.89	**<0.001**	5.01	1.19	4.82	1.36	0.279	0.269	0.268
HDL-c, mmol/L	1.26	0.23	1.36	0.28	**<0.001**	1.38	0.33	1.36	0.27	0.515	0.118	0.972
LDL-c, mmol/L	2.98	0.65	3.32	0.73	**<0.001**	3.21	1.02	3.18	1.08	0.693	0.316	0.570
LDL-c/HDL-c	2.46	0.78	2.55	0.80	0.120	2.41	0.83	2.41	0.89	0.959	0.815	0.554
Triglycerides, mmol/L	1.06	0.45	1.08	0.52	0.776	0.91	0.46	0.93	0.46	0.652	0.251	0.298
Glucose, mmol/L	4.8	0.51	4.87	0.56	0.336	4.96	0.45	5.03	0.39	0.355	0.251	0.253
Insulin, µIU/mL *	2.3	1.0; 4.2	3.1	1.0; 5.5	0.119	3.0	1.0; 3.9	3.6	1.0; 5.1	0.762	0.925	0.857
HOMA-IR	0.8	0.8	1.0	1.0	0.105	0.8	0.9	1.1	1.3	0.403	0.908	0.837
hs-CRP, mg/L *	1.3	1.0; 1.5	1.5	1.2; 2.3	0.477	0.8	0.6; 1.4	1.0	0.6; 1.7	0.447	0.598	0.163
Lp(a), mg/dL *	12.2	7.6; 20.0	14.0	7.2; 20.2	0.931	15.5	9.1; 27.4	15.9	10.1; 26.6	0.548	0.710	0.491
Homocysteine, µmol/L	8.5	3.1	8.4	3.4	0.893	7.9	2.1	7.8	2.2	0.856	0.438	0.456
Folic acid, µ/L	13.3	4.5	13.6	5.1	0.650	17.3	5.5	16.7	5.2	0.448	**0.006**	**0.039**
Sirtuin-1, ng/mL	0.40	0.13	0.49	0.16	**0.003**	0.51	0.10	0.58	0.14	**0.004**	**0.003**	**0.043**

Data are presented in mean and standard deviation (SD) or median and interquartile range (IQR) depending on the variable’s distribution. *: Nonparametric variables. Baseline *p*: comparison of baseline variables between groups. Post-treatment *p*: comparison of variables at the end of the study between groups. Bold: highlight the statistical significance.

**Table 2:** Comparison of percentage changes between groups.

	Change %				
	Blended Coffee Group		Arabica Coffee Group		*p*
	*n* = 33		*n* = 20		
Variables	% Mean Change	SD	% Mean Change	SD	
Body weight, kg	−0.2	1.6	−0.3	1.3	0.738
Body mass index, kg/m^2^	0.4	1.8	−0.4	1.3	0.092
Total cholesterol, mmol/L	10.7	14.8	−3.5	15.3	**0.002**
HDL-c, mmol/L	8.0	11.6	−0.6	10.6	**0.009**
LDL-c, mmol/L	12.7	18.3	−1.1	10.3	**0.003**
LDL-c/HDL-c	4.6	13.6	−0.1	2.08	0.183
Triglycerides, mmol/L	9.8	47.9	9.4	37.8	0.975
Glucose, mmol/L	1.7	8.3	1.8	6.9	0.970
Insulin, µIU/mL *	0.0	0.0; 54.9	0.0	−8.75; 21.4	0.448
HOMA-IR	7.9	−0.7; 58.9	4.1	−8.75; 23.0	0.509
hs-CRP, mg/L *	13.3	−12.3; 56.9	10.0	−7.6; 39.3	0.985
Lp(a), mg/dL	3.6	29.9	−5.2	17.2	0.233
Homocysteine, µmol/L	1.9	29.5	1.5	22.2	0.956
Folic acid, µ/L	3.4	21.6	−0.8	20.3	0.487
Sirtuin-1, ng/mL *	15.1	6.5; 29.5	15.8	5.5; 24.5	0.846

Data are presented in mean and standard deviation (SD) or median and interquartile range (IQR) depending on the variable’s distribution. *: Nonparametric variables. Bold: highlight the statistical significance.

**Table 3:** Effects of interventions on outcomes.

	Tests of Fixed Effects *	
Outcome	F	*p*-Value
*Sirt-1*		
Intercept	938.542	**<0.001**
Time	17.641	**<0.001**
Coffee type	9.071	**0.004**
Time*Coffee type	0.133	0.717
*Total Cholesterol*		
Intercept	69.891	**<0.001**
Time	2.270	0.138
Coffee type	0.192	0.663
Time*Coffee type	12.399	**0.001**
*LDL-c*		
Intercept	28.211	**<0.001**
Time	5.950	**0.018**
Coffee type	0.039	0.844
Time*Coffee type	8.992	**0.004**
*HDL-c*		
Intercept	1382.005	**<0.001**
Time	3.224	0.078
Coffee type	0.832	0.366
Time*Coffee type	8.495	**0.005**

*: Linear mixed models adjusted by age and sex. Bold: highlight the statistical significance.

**Table 4:** Effects of different coffee powders on Sirt-1 and cholesterol adjusted by age and sex.

	Estimate Fixed Effects					Pairwise Comparisons			
				95% CI		Mean Effect		95% CI	
Outcome	Estimate	SE	*t*	Lower	Upper		SE	Lower	Upper
*Sirt-1*, ng/mL									
Intercept	0.584	0.033	17.426	**0.516**	**0.651**				
Time (pre vs. post)	−0.080	0.033	−2.425	**−0.147**	**−0.014**	0.088	0.021	**0.046**	**0.130**
Coffee type (Arabica vs. blend)	−0.090	0.042	−2.112	**−0.175**	**−0.005**	0.097	0.032	**0.032**	**0.162**
Time*Coffee type	−0.015	0.042	−0.364	-0.099	0.069				
*Total Cholesterol*, mmol/L									
Intercept	3.469	0.482	7.199	**2.499**	**4.439**				
Time (pre vs. post)	0.186	0.146	1.277	−0.106	0.479	0.139	0.092	−0.046	0.325
Coffee type (Arabica vs. blend)	0.434	0.281	1.548	−0.129	0.998	−0.109	0.249	−0.608	0.390
Time*Coffee type	−0.651	0.185	−3.521	**−1.022**	**−0.280**				
*LDL-c*, mmol/L									
Intercept	1.871	0.397	4.719	**1.075**	**2.668**				
Time (pre vs. post)	0.035	0.098	0.355	−0.163	0.232	0.152	0.062	**0.027**	**0.277**
Coffee type (Arabica vs. blend)	0.227	0.221	1.027	−0.217	0.671	−0.040	0.203	−0.447	0.367
Time*Coffee type	−0.374	0.125	−2.999	**−0.625**	**−0.124**				
*HDL-c*, mmol/L									
Intercept	1.412	0.057	24.627	**1.297**	**1.527**				
Time (pre vs. post)	0.023	0.033	0.709	−0.043	0.089	0.037	0.021	−0.004	0.079
Coffee type (Arabica vs. blend)	−0.001	0.070	−0.008	−0.140	0.139	0.061	0.067	−0.073	0.196
Time*Coffee type	−0.121	0.042	−2.915	**−0.205**	**−0.038**				

Bold: highlight the statistical significance.

## Data Availability

Not applicable.

## Appendix A

**Table A1:** Differences in chemical composition (g per 100 g of sample in dry base) of coffee species. Adapted from De Souza, R.M.N.; Benassi, M.T. [27].

	Coffee Species			
Chemical Composition	Arabica		Robusta	
	Mean	SD	Mean	SD
Nicotinic acid	0.03	0.01	0.02	0.00
Trigonelline	0.49	0.20	0.22	0.14
5-CQA	0.29	0.13	0.21	0.15
Caffeine	1.22	0.09	2.01	0.03
Kahweol	0.82	0.10	n.d.	n.d.
Cafestol	0.37	0.08	0.21	0.03

Data are presented in mean and standard deviation (SD). 5-CQA: 5-caffeoylquinic acid.

## References

[1] Kane A.E., Sinclair D.A. (2018). Sirtuins and NAD+ in the development and treatment of metabolic and cardiovascular diseases. Circ. Res..

[2] Kwon H.S., Ott M. (2008). The ups and downs of SIRT1. Trends Biochem. Sci..

[3] Beher D., Wu J., Cumine S., Kim K.W., Lu S.C., Atangan L., Wang M. (2009). Resveratrol is not a direct activator of sirt1 enzyme activity. Chem. Biol. Drug Des..

[4] Palacios J.A., Herranz D., De Bonis M.L., Velasco S., Serrano M., Blasco M.A. (2010). SIRT1 contributes to telomere maintenance and augments global homologous recombination. J. Cell Biol..

[5] Weindruch R., Chia D., Barnett E.V., Walford R.L. (1982). Dietary restriction in mice beginning at 1 year of age: Effects on serum immune complex levels. Age.

[6] Mansur A.P., Roggerio A., Goes M.F.S., Avakian S.D., Leal D.P., Maranhão R.C., Strunz C.M.C. (2017). Serum concentrations and gene expression of sirtuin 1 in healthy and slightly overweight subjects after caloric restriction or resveratrol supplementation: A randomized trial. Int. J. Cardiol..

[7] Roggerio A., Cassaro Strunz C.M., Pacanaro A.P., Leal D.P., Takada J.Y., Avakian S.D., Mansur A.D. (2018). Gene expression of sirtuin-1 and endogenous secretory receptor for advanced glycation end products in healthy and slightly overweight subjects after caloric restriction and resveratrol administration. Nutrients.

[8] Gonçalinho G.H.F., Roggerio A., Goes M.F.d.S., Avakian S.D., Leal D.P., Strunz C.M.C., Mansur A.D. (2021). Comparison of Resveratrol Supplementation and Energy Restriction Effects on Sympathetic Nervous System Activity and Vascular Reactivity: A Randomized Clinical Trial. Molecules.

[9] Timmers S., Konings E., Bilet L., Houtkooper R.H., Van De Weijer T., Goossens G.H., Hoeks J., Van Der Krieken S., Ryu D., Kersten S. (2011). Calorie restriction-like effects of 30 days of resveratrol supplementation on energy metabolism and metabolic profile in obese humans. Cell Metab..

[10] Gomes A.P., Price N.L., Ling A.J.Y., Moslehi J.J., Montgomery M.K., Rajman L., White J.P., Teodoro J.S., Wrann C.D., Hubbard B.P. (2013). Declining NAD+ induces a pseudohypoxic state disrupting nuclear-mitochondrial communication during aging. Cell.

[11] Vachharajani V.T., Liu T., Wang X., Hoth J.J., Yoza B.K., McCall C.E. (2016). Sirtuins Link Inflammation and Metabolism. J. Immunol. Res..

[12] Bonkowski M.S., Sinclair D.A. (2016). Slowing ageing by design: The rise of NAD+ and sirtuin-activating compounds. Nat. Rev. Mol. Cell Biol..

[13] de Boer V.C.J., de Goffau M.C., Arts I.C.W., Hollman P.C.H., Keijer J. (2006). SIRT1 stimulation by polyphenols is affected by their stability and metabolism. Mech. Ageing Dev..

[14] Ding M., Bhupathiraju S.N., Satija A., Van Dam R.M., Hu F.B. (2014). Long-term coffee consumption and risk of cardiovascular disease: A systematic review and a dose-response meta-analysis of prospective cohort studies. Circulation.

[15] Poole R., Kennedy O.J., Roderick P., Fallowfield J.A., Hayes P.C., Parkes J. (2017). Coffee consumption and health: Umbrella review of meta-analyses of multiple health outcomes. BMJ.

[16] Hu G.L., Wang X., Zhang L., Qiu M.H. (2019). The sources and mechanisms of bioactive ingredients in coffee. Food Funct..

[17] Du Y., Lv Y., Zha W., Hong X., Luo Q. (2020). Effect of coffee consumption on dyslipidemia: A meta-analysis of randomized controlled trials. Nutr. Metab. Cardiovasc. Dis..

[18] Ludwig I.A., Mena P., Calani L., Cid C., Del Rio D., Lean M.E.J., Crozier A. (2014). Variations in caffeine and chlorogenic acid contents of coffees: What are we drinking?. Food Funct..

[19] Wongsa P., Khampa N., Horadee S., Chaiwarith J., Rattanapanone N. (2019). Quality and bioactive compounds of blends of Arabica and Robusta spray-dried coffee. Food Chem..

[20] Mensink R.P., Lebbink W.J., Lobbezoo I.E., der Wouw M.P.W.V., Zock P.L., Katan M.B. (1995). Diterpene composition of oils from Arabica and Robusta coffee beans and their effects on serum lipids in man. J. Intern. Med..

[21] Ciaramelli C., Palmioli A., Airoldi C. (2019). Coffee variety, origin and extraction procedure: Implications for coffee beneficial effects on human health. Food Chem..

[22] Bravo J., Arbillaga L., De Peña M.P., Cid C. (2013). Antioxidant and genoprotective effects of spent coffee extracts in human cells. Food Chem. Toxicol..

[23] Bakuradze T., Lang R., Hofmann T., Stiebitz H., Bytof G., Lantz I., Baum M., Eisenbrand G., Janzowski C. (2010). Antioxidant effectiveness of coffee extracts and selected constituents in cell-free systems and human colon cell lines. Mol. Nutr. Food Res..

[24] Chandrasekar V., Viswanathan R. (1999). Physical and thermal properties of soybean. J. Agric. Engng Res.

[25] Eira M.T.S., Amaral Da Silva E.A., De Castro R.D., Dussert S., Walters C., Bewley J.D., Hilhorst H.W.M. (2006). Coffee seed physiology. Braz. J. Plant Physiol..

[26] Cagliani L.R., Pellegrino G., Giugno G., Consonni R. (2013). Quantification of Coffea arabica and Coffea canephora var. robusta in roasted and ground coffee blends. Talanta.

[27] De Souza R.M.N., Benassi M.T. (2012). Discrimination of commercial roasted and ground coffees according to chemical composition. J. Braz. Chem. Soc..

[28] He X., Zheng J., Liu C. (2019). Low serum level of sirtuin 1 predicts coronary atherosclerosis plaques during computed tomography angiography among an asymptomatic cohort. Coron. Artery Dis..

[29] Rebollo-Hernanz M., Zhang Q., Aguilera Y., Martín-Cabrejas M.A., Gonzalez de Mejia E. (2019). Phenolic compounds from coffee by-products modulate adipogenesis-related inflammation, mitochondrial dysfunction, and insulin resistance in adipocytes, via insulin/PI3K/AKT signaling pathways. Food Chem. Toxicol..

[30] Ho L., Varghese M., Wang J., Zhao W., Chen F., Knable L.A., Ferruzzi M., Pasinetti G.M. (2012). Dietary supplementation with decaffeinated green coffee improves diet-induced insulin resistance and brain energy metabolism in mice. Nutr. Neurosci..

[31] Ommati M.M., Farshad O., Mousavi K., Khalili M., Jamshidzadeh A., Heidari R. (2020). Chlorogenic acid supplementation improves skeletal muscle mitochondrial function in a rat model of resistance training. Biologia.

[32] de Oliveira M.R., de Souza I.C.C., Fürstenau C.R. (2020). Mitochondrial Protection Promoted by the Coffee Diterpene Kahweol in Methylglyoxal-Treated Human Neuroblastoma SH-SY5Y Cells. Neurotox. Res..

[33] Fürstenau C.R., de Souza I.C.C., de Oliveira M.R. (2019). The effects of kahweol, a diterpene present in coffee, on the mitochondria of the human neuroblastoma SH-SY5Y cells exposed to hydrogen peroxide. Toxicol. In Vitro.

[34] Lagouge M., Argmann C., Gerhart-Hines Z., Meziane H., Lerin C., Daussin F., Messadeq N., Milne J., Lambert P., Elliott P. (2006). Resveratrol Improves Mitochondrial Function and Protects against Metabolic Disease by Activating SIRT1 and PGC-1α. Cell.

[35] van Dam R.M., Hu F.B., Willett W.C. (2020). Coffee, Caffeine, and Health. N. Engl. J. Med..

[36] Torres-collado L., Compañ-gabucio L.M., González-palacios S., Notario-barandiaran L., Oncina-cánovas A., Vioque J., García-de la Hera M. (2021). Coffee consumption and all-cause, cardiovascular, and cancer mortality in an adult mediterranean population. Nutrients.

[37] Gunter M.J., Murphy N., Cross A.J., Dossus L., Dartois L., Fagherazzi G., Kaaks R., Kühn T., Boeing H., Aleksandrova K. (2017). Coffee drinking and mortality in 10 European countries: A multinational cohort study. Ann. Intern. Med..

[38] Tsai K.L., Hung C.H., Chan S.H., Hsieh P.L., Ou H.C., Cheng Y.H., Chu P.M. (2018). Chlorogenic Acid Protects Against oxLDL-Induced Oxidative Damage and Mitochondrial Dysfunction by Modulating SIRT1 in Endothelial Cells. Mol. Nutr. Food Res..

[39] Hada Y., Uchida H.A., Otaka N., Onishi Y., Okamoto S., Nishiwaki M., Takemoto R., Takeuchi H., Wada J. (2020). The protective effect of chlorogenic acid on vascular senescence via the Nrf2/HO-1 pathway. Int. J. Mol. Sci..

[40] Yang L., Wei J., Sheng F., Li P. (2019). Attenuation of Palmitic Acid–Induced Lipotoxicity by Chlorogenic Acid through Activation of SIRT1 in Hepatocytes. Mol. Nutr. Food Res..

[41] Wanke V., Cameroni E., Uotila A., Piccolis M., Urban J., Loewith R., De Virgilio C. (2008). Caffeine extends yeast lifespan by targeting TORC1. Mol. Microbiol..

[42] Tao L., Zhang W., Zhang Y., Zhang M., Zhang Y., Niu X., Zhao Q., Liu Z., Li Y., Diao A. (2021). Caffeine promotes the expression of telomerase reverse transcriptase to regulate cellular senescence and aging. Food Funct..

[43] Guo W., Qian L., Zhang J., Zhang W., Morrison A., Hayes P., Wilson S., Chen T., Zhao J. (2011). Sirt1 overexpression in neurons promotes neurite outgrowth and cell survival through inhibition of the mTOR signaling. J. Neurosci. Res..

[44] Amano H., Chaudhury A., Rodriguez-Aguayo C., Lu L., Akhanov V., Catic A., Popov Y.V., Verdin E., Johnson H., Stossi F. (2019). Telomere Dysfunction Induces Sirtuin Repression that Drives Telomere-Dependent Disease. Cell Metab..

[45] Xu H., Gan C., Gao Z., Huang Y., Wu S., Zhang D., Wang X., Sheng J. (2020). Caffeine Targets SIRT3 to Enhance SOD2 Activity in Mitochondria. Front. Cell Dev. Biol..

[46] Zhang S.J., Li Y.F., Wang G.E., Tan R.R., Tsoi B., Mao G.W., Zhai Y.J., Cao L.F., Chen M., Kurihara H. (2015). Caffeine ameliorates high energy diet-induced hepatic steatosis: Sirtuin 3 acts as a bridge in the lipid metabolism pathway. Food Funct..

[47] Urgert R., Katan M.B. (1996). The cholesterol-raising factor from coffee beans. J. R. Soc. Med..

[48] Godos J., Pluchinotta F.R., Marventano S., Buscemi S., Volti G.L., Galvano F., Grosso G. (2014). Coffee components and cardiovascular risk: Beneficial and detrimental effects. Int. J. Food Sci. Nutr..

[49] Superko H.R., Bortz W., Williams P.T., Albers J.J., Wood P.D. (1991). Caffeinated and decaffeinated coffee effects on plasma lipoprotein cholesterol, apolipoproteins, and lipase activity: A controlled, randomized trial. Am. J. Clin. Nutr..

[50] Uto-Kondo H., Ayaori M., Ogura M., Nakaya K., Ito M., Suzuki A., Takiguchi S.I., Yakushiji E., Terao Y., Ozasa H. (2010). Coffee consumption enhances high-density lipoprotein-mediated cholesterol efflux in macrophages. Circ. Res..

[51] Christensen B., Mosdol A., Retterstol L., Landaas S., Thelle D.S. (2001). Abstention from filtered coffee reduces the concentrations of plasma homocysteine and serum cholesterol—A randomized controlled trial. Am. J. Clin. Nutr..

[52] Corrêa T.A.F., Rogero M.M., Mioto B.M., Tarasoutchi D., Tuda V.L., César L.A.M., Torres E.A.F.S. (2013). Paper-filtered coffee increases cholesterol and inflammation biomarkers independent of roasting degree: A clinical trial. Nutrition.

[53] Muzykiewicz-Szymańska A., Nowak A., Wira D., Klimowicz A. (2021). The Effect of Brewing Process Parameters on Antioxidant Activity and Caffeine Content in Infusions of Roasted and Unroasted Arabica Coffee Beans Originated from Different Countries. Molecules.

[54] Fears R. (1978). The hypercholesterolaemic effect of caffeine in rats fed on diets with and without supplementary cholesterol. Br. J. Nutr..

[55] Hu S., Liu K., Luo H., Xu D., Chen L., Zhang L., Wang H. (2019). Caffeine programs hepatic SIRT1-related cholesterol synthesis and hypercholesterolemia via A2AR/cAMP/PKA pathway in adult male offspring rats. Toxicology.

[56] Hung C.H., Chan S.H., Chu P.M., Tsai K.L. (2015). Homocysteine facilitates LOX-1 activation and endothelial death through the PKCβ and SIRT1/HSF1 mechanism: Relevance to human hyperhomocysteinaemia. Clin. Sci..

[57] Chen Y., Liu H., Wang X., Zhang H., Liu E., Su X. (2017). Homocysteine up-regulates endothelin type A receptor in vascular smooth muscle cells through Sirt1/ERK1/2 signaling pathway. Microvasc. Res..

[58] Chen Y., Liu H., Zhang H., Liu E., Xu C.B., Su X. (2016). The sirt1/NF-kB signaling pathway is involved in regulation of endothelin type B receptors mediated by homocysteine in vascular smooth muscle cells. Biomed. Pharmacother..

[59] Ganguly P., Alam S.F. (2015). Role of homocysteine in the development of cardiovascular disease. Nutr. J..

[60] Urgert R., Van Vliet T., Zock P.L., Katan M.B. (2000). Heavy coffee consumption and plasma homocysteine: A randomized controlled trial in healthy volunteers. Am. J. Clin. Nutr..

[61] Verhoef P., Pasman W.J., Van Vliet T., Urgert R., Katan M.B. (2002). Contribution of caffeine to the homocysteine-raising effect of coffee: A randomized controlled trial in humans. Am. J. Clin. Nutr..

[62] Mursu J., Voutilainen S., Nurmi T., Alfthan G., Virtanen J.K., Rissanen T.H., Happonen P., Nyyssönen K., Kaikkonen J., Salonen R. (2005). The effects of coffee consumption on lipid peroxidation and plasma total homocysteine concentrations: A clinical trial. Free Radic. Biol. Med..

[63] Gonçalinho G.H.F., Sampaio G.R., Soares-Freitas R.A.M., Damasceno N.R.T. (2021). Omega-3 Fatty Acids in Erythrocyte Membranes as Predictors of Lower Cardiovascular Risk in Adults without Previous Cardiovascular Events. Nutrients.

[64] Kida Y., Goligorsky M.S. (2016). Sirtuins, Cell Senescence, and Vascular Aging. Can. J. Cardiol..

[65] Zhong Y., Chen A.F., Zhao J., Gu Y.J., Fu G.X. (2016). Serum levels of cathepsin D, sirtuin1, and endothelial nitric oxide synthase are correlatively reduced in elderly healthy people. Aging Clin. Exp. Res..

[66] Gok O., Karaali Z., Ergen A., Ekmekci S.S., Abaci N. (2019). Serum sirtuin 1 protein as a potential biomarker for type 2 diabetes: Increased expression o sirtuin 1 and the correlation with microRNAs. J. Res. Med. Sci..

[67] Kumar R., Chaterjee P., Sharma P.K., Singh A.K., Gupta A., Gill K., Tripathi M., Dey A.B., Dey S. (2013). Sirtuin1: A Promising Serum Protein Marker for Early Detection of Alzheimer’s Disease. PLoS ONE.

